# Supplementary material for: Development of a Sensitive Digital Droplet PCR Screening Assay for the Detection of GPR126 Non-Coding Mutations in Bladder Cancer Urine Liquid Biopsies
Source: Biomedicines. 2023 Feb 8;11(2):495. doi: 10.3390/biomedicines11020495 (PMC9953558; doi:10.3390/biomedicines11020495)
Supplement: Supplementary file 1 [file biomedicines-11-00495-s001.zip › Figure S1_Jain.pdf]

GPR126 6th intron Synthetic DNA construct wild type DNA clone

ATCACCAGGGACCCAAAACCCTGAGAAAAGAAACCCACATAGTTCATTTTTTCTTGGCTTCAGAGGTTACATCAATT
TCCTCTGTAGGACATTTAAATTGGAACTATGGAATATTTTACATGAAACATAGATATAAATTTCAGTAGGGTTATAA
AGACATCGATTCAGATAGTGTTTATGGTTATTCTGATAGTTACACCAAGGAGATTTATGATGGAGCAAAAATTCCAG
CAACCCCCAAGAAAGCAACAGCAGCTGAAATTTCCAGTGCATATTTCACATGGACTCTAGCCAGTAGATGGGAATAT
TATAATTCTAACTAAGTATTTCACTCTTTGTATGAACATACAAAGAGCCTCTGTGGTATTCTCCCTCCAGGATTGAC
ACATCCTCTACTGTCCAAGAACATTTATACGGTGCTTCAGAGTCCTTGACTTGGTGGAGTGGAGCTGTATTCACTCA
ATCATGTCATTTCTGACATTCTTGAAGAAAGCCTTGTTTAGGCCATCTCTCTCTGCGTCATAACCTTCATTGGAGTT
TTAACAAAGCAAGCAAATGTTAGAATCAGTTAAAATGGTCTGTCTTGACCACATTAATGAAAAAAATCTAATTCAGA
TAATTCTTAATCAAAATGTCATCAGCGTTTAAAAATATTCAGGTCATTATATGACAGAAATATTCTCAAAATTGTGT
TCTCAAATTTGTGTACCATCTTAACAATTGTTATTTTTACCATGTCTCTGAACCACCTATAC

```
-=-=-=-=
M13dir primer (direct strand):
    FILE NAME     58        67         77         87         97        107
117
    5D.SEQ
GCGGAGACATTATCACCAGGGACCCAAAACCCTGAGAAAAGAAACCCACATAGTTCATTT

|||||||||||||||||||||||||||||||||||||||||||||||||
    INI.SEQ
...........ATCACCAGGGACCCAAAACCCTGAGAAAAGAAACCCACATAGTTCATTT
                 -10        -1          9         19         29          39
49

    FILE NAME    118       127        137        147        157        167
177
    5D.SEQ
TTTCTTGGCTTCAGAGGTTACATCAATTTCCTCTGTAGGACATTTAAATTGGAACTATGG

||||||||||||||||||||||||||||||||||||||||||||||||||||||||||||
    INI.SEQ
TTTCTTGGCTTCAGAGGTTACATCAATTTCCTCTGTAGGACATTTAAATTGGAACTATGG
                  50        59         69         79         89          99
109

    FILE NAME    178       187        197        207        217        227
237
    5D.SEQ
AATATTTTACATGAAACATAGATATAAATTTCAGTAGGGTTATAAAGACATCGATTCAGA

||||||||||||||||||||||||||||||||||||||||||||||||||||||||||||
    INI.SEQ
AATATTTTACATGAAACATAGATATAAATTTCAGTAGGGTTATAAAGACATCGATTCAGA
                 110       119        129        139        149        159
169

    FILE NAME    238       247        257        267        277        287
297
    5D.SEQ
TAGTGTTTATGGTTATTCTGATAGTTACACCAAGGAGATTTATGATGGAGCAAAAATTCC

||||||||||||||||||||||||||||||||||||||||||||||||||||||||||||
    INI.SEQ
TAGTGTTTATGGTTATTCTGATAGTTACACCAAGGAGATTTATGATGGAGCAAAAATTCC
                 170       179        189        199        209        219
229

    FILE NAME    298       307        317        327        337        347
357
    5D.SEQ
AGCAACCCCCAAGAAAGCAACAGCAGCTGAAATTTCCAGTGCATATTTCACATGGACTCT

||||||||||||||||||||||||||||||||||||||||||||||||||||||||||||
```

```
    INI.SEQ
AGCAACCCCCAAGAAAGCAACAGCAGCTGAAATTTCCAGTGCATATTTCACATGGACTCT
                 230      239       249       259       269       279
289

    FILE NAME    358      367       377       387       397       407
417
    5D.SEQ
AGCCAGTAGATGGGAATATTATAATTCTAACTAAGTATTTCACTCTTTGTATGAACATAC

||||||||||||||||||||||||||||||||||||||||||||||||||||||||||||
    INI.SEQ
AGCCAGTAGATGGGAATATTATAATTCTAACTAAGTATTTCACTCTTTGTATGAACATAC
                 290      299       309       319       329       339
349

    FILE NAME    418      427       437       447       457       467
477
    5D.SEQ
AAAGAGCCTCTGTGGTATTCTCCCTCCAGGATTGACACATCCTCTACTGTCCAAGAACAT

||||||||||||||||||||||||||||||||||||||||||||||||||||||||||||
    INI.SEQ
AAAGAGCCTCTGTGGTATTCTCCCTCCAGGATTGACACATCCTCTACTGTCCAAGAACAT
                 350      359       369       379       389       399
409

    FILE NAME    478      487       497       507       517       527
537
    5D.SEQ
TTATACGGTGCTTCAGAGTCCTTGACTTGGTGGAGTGGAGCTGTATTCACTCAATCATGT

||||||||||||||||||||||||||||||||||||||||||||||||||||||||||||
    INI.SEQ
TTATACGGTGCTTCAGAGTCCTTGACTTGGTGGAGTGGAGCTGTATTCACTCAATCATGT
                 410      419       429       439       449       459
469

    FILE NAME    538      547       557       567       577       587
597
    5D.SEQ
CATTTCTGACATTCTTGAAGAAAGCCTTGTTTAGGCCATCTCTCTCTGCGTCATAACCTT

||||||||||||||||||||||||||||||||||||||||||||||||||||||||||||
    INI.SEQ
CATTTCTGACATTCTTGAAGAAAGCCTTGTTTAGGCCATCTCTCTCTGCGTCATAACCTT
                 470      479       489       499       509       519
529

    FILE NAME    598      607       617       627       637       647
657
    5D.SEQ
CATTGGAGTTTTAACAAAGCAAGCAAATGTTAGAATCAGTTAAAATGGTCTGTCTTGACC

||||||||||||||||||||||||||||||||||||||||||||||||||||||||||||
    INI.SEQ
CATTGGAGTTTTAACAAAGCAAGCAAATGTTAGAATCAGTTAAAATGGTCTGTCTTGACC
                 530      539       549       559       569       579
589

    FILE NAME    658      667       677       687       697       707
717
    5D.SEQ
ACATTAATGAAAAAAATCTAATTCAGATAATTCTTAATCAAAATGTCATCAGCGTTTAAA
```

```
||||||||||||||||||||||||||||||||||||||||||||||||||||||||||||
    INI.SEQ
ACATTAATGAAAAAAATCTAATTCAGATAATTCTTAATCAAAATGTCATCAGCGTTTAAA
                 590      599       609       619       629       639
649
-==-
M13rev primer (complement):
    FILE NAME    820      829       839       849       859       869
879
    5R.SEQ
GCAAATGTTAGAATCAGTTAAAATGGTCTGTCTTGACCACATTAATGAAAAAAATCTAAT

||||||||||||||||||||||||||||||||||||||||||||||||||||||||||||
    INI.SEQ
GCAAATGTTAGAATCAGTTAAAATGGTCTGTCTTGACCACATTAATGAAAAAAATCTAAT
                 552      561       571       581       591       601
611

    FILE NAME    880      889       899       909       919       929
939
    5R.SEQ
TCAGATAATTCTTAATCAAAATGTCATCAGCGTTTAAAAATATTCAGGTCATTATATGAC

||||||||||||||||||||||||||||||||||||||||||||||||||||||||||||
    INI.SEQ
TCAGATAATTCTTAATCAAAATGTCATCAGCGTTTAAAAATATTCAGGTCATTATATGAC
                 612      621       631       641       651       661
671

    FILE NAME    940      949       959       969       979       989
999
    5R.SEQ
AGAAATATTCTCAAAATTGTGTTCTCAAATTTGTGTACCATCTTAACAATTGTTATTTTT

||||||||||||||||||||||||||||||||||||||||||||||||||||||||||||
    INI.SEQ
AGAAATATTCTCAAAATTGTGTTCTCAAATTTGTGTACCATCTTAACAATTGTTATTTTT
                 672      681       691       701       711       721
731

    FILE NAME   1000     1009      1019
    5R.SEQ         ACCATGTCTCTGAACCACCTATAC
                   ||||||||||||||||||||||||
    INI.SEQ        ACCATGTCTCTGAACCACCTATAC
                 732      741       751
```

GPR126 6th intron Synthetic DNA construct “chr. 6: 142,706,206; G/A substitution” clone

```
ATCACCAGGGACCCAAAACCCTGAGAAAAGAAACCCACATAGTTCATTTTTTCTTGGCTTCAGAGGTTACATCAATT
TCCTCTGTAGGACATTTAAATTGGAACTATGGAATATTTTACATGAAACATAGATATAAATTTCAGTAGGGTTATAA
AGACATCGATTCAGATAGTGTTTATGGTTATTCTGATAGTTACACCAAGGAGATTTATGATGGAGCAAAAATTCCAG
CAACCCCCAAGAAAGCAACAGCAGCTGAAATTTCCAGTGCATATTTCACATGGACTCTAGCCAGTAGATGGGAATAT
TATAATTCTAACTAAGTATTTCACTCTTTGTATGAACATACAAAGAGCCTCTGTGGTATTCTCCCTCCAGGATTGAC
ACATCCTCTACTGTCCAAGAACATTTATACGGTGCTTCAGAGTCCTTGACTTGGTGGAGTGGAGCTGTATTCACTCA
ATCATGTCATTTCTGACATTCTTGAAGAAAGCCTTGTTTAGGCCATCTCTCTCTGCGTCATAACCTTCATTGGAGTT
TTAACAAAGCAAGCAAATGTTAGAATCAGTTAAAATGGTCTGTCTTGACCACATTAATGAAAAAAATCTAATTCAGA
TAATTCTTAATCAAAATGTCATCAGCGTTTAAAAATATTCAGGTCATTATATGACAGAAATATTCTCAAAATTGTGT
TCTCAAATTTGTGTACCATCTTAACAATTGTTATTTTTACCATGTCTCTGAACCACCTATAC
=-===================
M13dir primer (direct strand):
    FILE NAME      74        83         93        103        113        123
133
    G1-2_M~2.SEQ
GCTCTCCCATATGGTCGACCTGCAGGTCGAATTCAGACATATCACCAGGGACCCAAAACC

||||||||||||||||||||
    INI.SEQ
........................................ATCACCAGGGACCCAAAACC
                 -39       -30        -20        -10          0         10
20

    FILE NAME     134       143        153        163        173        183
193
    G1-2_M~2.SEQ
CTGAGAAAAGAAACCCACATAGTTCATTTTTTCTTGGCTTCAGAGGTTACATCAATTTCC

||||||||||||||||||||||||||||||||||||||||||||||||||||||||||||
    INI.SEQ
CTGAGAAAAGAAACCCACATAGTTCATTTTTTCTTGGCTTCAGAGGTTACATCAATTTCC
                  21        30         40         50         60         70
80

    FILE NAME     194       203        213        223        233        243
253
    G1-2_M~2.SEQ
TCTGTAGGACATTTAAATTGGAACTATGGAATATTTTACATGAAACATAGATATAAATTT

||||||||||||||||||||||||||||||||||||||||||||||||||||||||||||
    INI.SEQ
TCTGTAGGACATTTAAATTGGAACTATGGAATATTTTACATGAAACATAGATATAAATTT
                  81        90        100        110        120        130
140

    FILE NAME     254       263        273        283        293        303
313
    G1-2_M~2.SEQ
CAGTAGGGTTATAAAGACATCGATTCAGATAGTGTTTATGGTTATTCTGATAGTTACACC

||||||||||||||||||||||||||||||||||||||||||||||||||||||||||||
    INI.SEQ
CAGTAGGGTTATAAAGACATCGATTCAGATAGTGTTTATGGTTATTCTGATAGTTACACC
                 141       150        160        170        180        190
200

    FILE NAME     314       323        333        343        353        363
373
    G1-2_M~2.SEQ
AAGGAGATTTATGATGGAGCAAAAATTCCAGCAACCCCCAAGAAAGCAACAGCAGCTGAA
```

```
||||||||||||||||||||||||||||||||||||||||||||||||||||||||||||
    INI.SEQ
AAGGAGATTTATGATGGAGCAAAAATTCCAGCAACCCCCAAGAAAGCAACAGCAGCTGAA
                 201      210       220       230       240       250
260

    FILE NAME    374      383       393       403       413       423
433
    G1-2_M~2.SEQ
ATTTCCAGTGCATATTTCACATGGACTCTAGCCAGTAGATGGGAATATTATAATTCTAAC

||||||||||||||||||||||||||||||||||||||||||||||||||||||||||||
    INI.SEQ
ATTTCCAGTGCATATTTCACATGGACTCTAGCCAGTAGATGGGAATATTATAATTCTAAC
                 261      270       280       290       300       310
320
-=-=
-=-=
M13rev primer (complement):
    FILE NAME       618
    G1-2_M~1.SEQ   TAAGTA
                   ||||||
    INI.SEQ        TAAGTA
                      326

    FILE NAME    619      628       638       648       658       668
678
    G1-2_M~1.SEQ
TTTCACTCTTTGTATAAACATACAAAGAGCCTCTGTGGTATTCTCCCTCCAGGATTGACA
                   |||||||||||||||
||||||||||||||||||||||||||||||||||||||||||||
    INI.SEQ
TTTCACTCTTTGTATGAACATACAAAGAGCCTCTGTGGTATTCTCCCTCCAGGATTGACA
                 327      336       346       356       366       376
386

    FILE NAME    679      688       698       708       718       728
738
    G1-2_M~1.SEQ
CATCCTCTACTGTCCAAGAACATTTATACGGTGCTTCAGAGTCCTTGACTTGGTGGAGTG

||||||||||||||||||||||||||||||||||||||||||||||||||||||||||||
    INI.SEQ
CATCCTCTACTGTCCAAGAACATTTATACGGTGCTTCAGAGTCCTTGACTTGGTGGAGTG
                 387      396       406       416       426       436
446

    FILE NAME    739      748       758       768       778       788
798
    G1-2_M~1.SEQ
GAGCTGTATTCACTCAATCATGTCATTTCTGACATTCTTGAAGAAAGCCTTGTTTAGGCC

||||||||||||||||||||||||||||||||||||||||||||||||||||||||||||
    INI.SEQ
GAGCTGTATTCACTCAATCATGTCATTTCTGACATTCTTGAAGAAAGCCTTGTTTAGGCC
                 447      456       466       476       486       496
506

    FILE NAME    799      808       818       828       838       848
858
    G1-2_M~1.SEQ
ATCTCTCTCTGCGTCATAACCTTCATTGGAGTTTTAACAAAGCAAGCAAATGTTAGAATC
```

```
||||||||||||||||||||||||||||||||||||||||||||||||||||||||||||
    INI.SEQ
ATCTCTCTCTGCGTCATAACCTTCATTGGAGTTTTAACAAAGCAAGCAAATGTTAGAATC
                 507      516       526       536       546       556
566

    FILE NAME    859      868       878       888       898       908
918
    G1-2_M~1.SEQ
AGTTAAAATGGTCTGTCTTGACCACATTAATGAAAAAAATCTAATTCAGATAATTCTTAA

||||||||||||||||||||||||||||||||||||||||||||||||||||||||||||
    INI.SEQ
AGTTAAAATGGTCTGTCTTGACCACATTAATGAAAAAAATCTAATTCAGATAATTCTTAA
                 567      576       586       596       606       616
626

    FILE NAME    919      928       938       948       958       968
978
    G1-2_M~1.SEQ
TCAAAATGTCATCAGCGTTTAAAAATATTCAGGTCATTATATGACAGAAATATTCTCAAA

||||||||||||||||||||||||||||||||||||||||||||||||||||||||||||
    INI.SEQ
TCAAAATGTCATCAGCGTTTAAAAATATTCAGGTCATTATATGACAGAAATATTCTCAAA
                 627      636       646       656       666       676
686

    FILE NAME    979      988       998      1008      1018      1028
1038
    G1-2_M~1.SEQ
ATTGTGTTCTCAAATTTGTGTACCATCTTAACAATTGTTATTTTTACCATGTCTCTGAAC

||||||||||||||||||||||||||||||||||||||||||||||||||||||||||||
    INI.SEQ
ATTGTGTTCTCAAATTTGTGTACCATCTTAACAATTGTTATTTTTACCATGTCTCTGAAC
                 687      696       706       716       726       736
746

    FILE NAME   1039     1048
    G1-2_M~1.SEQ   CACCTATAC
                   |||||||||
    INI.SEQ        CACCTATAC
                 747      756
```

GPR126 6th intron Synthetic DNA construct "chr. 6: 142,706,206; G/A substitution + chr. 6: 142,706,209; C/T substitution" clone

```
ATCACCAGGGACCCAAAACCCTGAGAAAAGAAACCCACATAGTTCATTTTTTCTTGGCTTCAGAGGTTACATCAATT
TCCTCTGTAGGACATTTAAATTGGAACTATGGAATATTTTACATGAAACATAGATATAAATTTCAGTAGGGTTATAA
AGACATCGATTCAGATAGTGTTTATGGTTATTCTGATAGTTACACCAAGGAGATTTATGATGGAGCAAAAATTCCAG
CAACCCCCAAGAAAGCAACAGCAGCTGAAATTTCCAGTGCATATTTCACATGGACTCTAGCCAGTAGATGGGAATAT
TATAATTCTAACTAAGTATTTCACTCTTTGTATGAACATACAAAGAGCCTCTGTGGTATTCTCCCTCCAGGATTGAC
ACATCCTCTACTGTCCAAGAACATTTATACGGTGCTTCAGAGTCCTTGACTTGGTGGAGTGGAGCTGTATTCACTCA
ATCATGTCATTTCTGACATTCTTGAAGAAAGCCTTGTTTAGGCCATCTCTCTCTGCGTCATAACCTTCATTGGAGTT
TTAACAAAGCAAGCAAATGTTAGAATCAGTTAAAATGGTCTGTCTTGACCACATTAATGAAAAAAATCTAATTCAGA
TAATTCTTAATCAAAATGTCATCAGCGTTTAAAAATATTCAGGTCATTATATGACAGAAATATTCTCAAAATTGTGT
TCTCAAATTTGTGTACCATCTTAACAATTGTTATTTTTACCATGTCTCTGAACCACCTATAC
=-===================
M13dir primer (direct strand):
    FILE NAME      19        28          38          48          58          68
78
    G2-8_M~2.SEQ
TGGGCCCGACGTCGCATGCTCCCGGCCGCCATGGCGGCCGCGGAGACATATCACCAGGGA

|||||||||||
    INI.SEQ
..................................................ATCACCAGGGA
                  -48       -39         -29         -19          -9           1
11

    FILE NAME      79        88          98         108         118         128
138
    G2-8_M~2.SEQ
CCCAAAACCCTGAGAAAAGAAACCCACATAGTTCATTTTTTCTTGGCTTCAGAGGTTACA

||||||||||||||||||||||||||||||||||||||||||||||||||||||||||||
    INI.SEQ
CCCAAAACCCTGAGAAAAGAAACCCACATAGTTCATTTTTTCTTGGCTTCAGAGGTTACA
                   12        21          31          41          51          61
71

    FILE NAME     139       148         158         168         178         188
198
    G2-8_M~2.SEQ
TCAATTTCCTCTGTAGGACATTTAAATTGGAACTATGGAATATTTTACATGAAACATAGA

||||||||||||||||||||||||||||||||||||||||||||||||||||||||||||
    INI.SEQ
TCAATTTCCTCTGTAGGACATTTAAATTGGAACTATGGAATATTTTACATGAAACATAGA
                   72        81          91         101         111         121
131

    FILE NAME     199       208         218         228         238         248
258
    G2-8_M~2.SEQ
TATAAATTTCAGTAGGGTTATAAAGACATCGATTCAGATAGTGTTTATGGTTATTCTGAT

||||||||||||||||||||||||||||||||||||||||||||||||||||||||||||
    INI.SEQ
TATAAATTTCAGTAGGGTTATAAAGACATCGATTCAGATAGTGTTTATGGTTATTCTGAT
                  132       141         151         161         171         181
191

    FILE NAME     259       268         278         288         298         308
318
    G2-8_M~2.SEQ
AGTTACACCAAGGAGATTTATGATGGAGCAAAAATTCCAGCAACCCCCAAGAAAGCAACA
```

```
||||||||||||||||||||||||||||||||||||||||||||||||||||||||||||
    INI.SEQ
AGTTACACCAAGGAGATTTATGATGGAGCAAAAATTCCAGCAACCCCCAAGAAAGCAACA
                 192      201       211       221       231       241
251

    FILE NAME    319      328       338       348       358       368
378
    G2-8_M~2.SEQ
GCAGCTGAAATTTCCAGTGCATATTTCACATGGACTCTAGCCAGTAGATGGGAATATTAT

||||||||||||||||||||||||||||||||||||||||||||||||||||||||||||
    INI.SEQ
GCAGCTGAAATTTCCAGTGCATATTTCACATGGACTCTAGCCAGTAGATGGGAATATTAT
                 252      261       271       281       291       301
311

    FILE NAME    379      388       398       408       418       428
438
    G2-8_M~2.SEQ
AATTCTAACTAAGTATTTCACTCTTTGTATGAATATACAAAGAGCCTCTGTGGTATTCTC
                   |||||||||||||||||||||||||||||||||
||||||||||||||||||||||||||
    INI.SEQ
AATTCTAACTAAGTATTTCACTCTTTGTATGAACATACAAAGAGCCTCTGTGGTATTCTC
                 312      321       331       341       351       361
371

    FILE NAME    439      448       458       468       478       488
498
    G2-8_M~2.SEQ
CCTCCAGGATTGACACATCCTCTACTGTCCAAGAACATTTATACGGTGCTTCAGAGTCCT

||||||||||||||||||||||||||||||||||||||||||||||||||||||||||||
    INI.SEQ
CCTCCAGGATTGACACATCCTCTACTGTCCAAGAACATTTATACGGTGCTTCAGAGTCCT
                 372      381       391       401       411       421
431

    FILE NAME    499      508       518       528       538       548
558
    G2-8_M~2.SEQ
TGACTTGGTGGAGTGGAGCTGTATTCACTCAATCATGTCATTTCTGACATTCTTGAAGAA

||||||||||||||||||||||||||||||||||||||||||||||||||||||||||||
    INI.SEQ
TGACTTGGTGGAGTGGAGCTGTATTCACTCAATCATGTCATTTCTGACATTCTTGAAGAA
                 432      441       451       461       471       481
491

    FILE NAME    559      568
    G2-8_M~2.SEQ   AGCCTTGTTTAGGCCAT
                   |||||||||||||||||
    INI.SEQ        AGCCTTGTTTAGGCCAT
                 492      501
-==-
M13rev primer (complement):
    FILE NAME    758      767       777       787       797       807
817
    G2-8_M~1.SEQ
CTCTCTCTGCGTCATAACCTTCATTGGAGTTTTAACAAAGCAAGCAAATGTTAGAATCAG

||||||||||||||||||||||||||||||||||||||||||||||||||||||||||||
```

```
    INI.SEQ
CTCTCTCTGCGTCATAACCTTCATTGGAGTTTTAACAAAGCAAGCAAATGTTAGAATCAG
                 510      519       529       539       549       559
569

    FILE NAME    818      827       837       847       857       867
877
    G2-8_M~1.SEQ
TTAAAATGGTCTGTCTTGACCACATTAATGAAAAAAATCTAATTCAGATAATTCTTAATC

||||||||||||||||||||||||||||||||||||||||||||||||||||||||||||
    INI.SEQ
TTAAAATGGTCTGTCTTGACCACATTAATGAAAAAAATCTAATTCAGATAATTCTTAATC
                 570      579       589       599       609       619
629

    FILE NAME    878      887       897       907       917       927
937
    G2-8_M~1.SEQ
AAAATGTCATCAGCGTTTAAAAATATTCAGGTCATTATATGACAGAAATATTCTCAAAA

|||||||||||||||||||||||||||||||||||||||||||||||||||||||||||
    INI.SEQ
AAAATGTCATCAGCGTTTAAAAATATTCAGGTCATTATATGACAGAAATATTCTCAAAA
                 630      639       649       659       669       679
689

    FILE NAME    938      947       957       967       977       987
997
    G2-8_M~1.SEQ
TTGTGTTCTCAAATTTGTGTACCATCTTAACAATTGTTATTTTTACCATGTCTCTGAACCACCTATAC

||||||||||||||||||||||||||||||||||||||||||||||||||||||||||||||||||||
    INI.SEQ
TTGTGTTCTCAAATTTGTGTACCATCTTAACAATTGTTATTTTTACCATGTCTCTGAACCACCTATAC
                 690      699       709       719       729       739
749
```

ATCACCAGGGACCCAAAACCCTGAGAAAAGAAACCCACATAGTTCATTTTTTCTTGGCTTCAGAGGTTACATCAATT
TCCTCTGTAGGACATTTAAATTGGAACTATGGAATATTTTACATGAAACATAGATATAAATTTCAGTAGGGTTATAA
AGACATCGATTCAGATAGTGTTTATGGTTATTCTGATAGTTACACCAAGGAGATTTATGATGGAGCAAAAATTCCAG
CAACCCCCAAGAAAGCAACAGCAGCTGAAATTTCCAGTGCATATTTCACATGGACTCTAGCCAGTAGATGGGAATAT
TATAATTCTAACTAAGTATTTCACTCTTTGTATGAACATACAAAGAGCCTCTGTGGTATTCTCCCTCCAGGATTGAC
ACATCCTCTACTGTCCAAGAACATTTATACGGTGCTTCAGAGTCCTTGACTTGGTGGAGTGGAGCTGTATTCACTCA
ATCATGTCATTTCTGACATTCTTGAAGAAAGCCTTGTTTAGGCCATCTCTCTCTGCGTCATAACCTTCATTGGAGTT
TTAACAAAGCAAGCAAATGTTAGAATCAGTTAAAATGGTCTGTCTTGACCACATTAATGAAAAAAATCTAATTCAGA
TAATTCTTAATCAAAATGTCATCAGCGTTTAAAAATATTCAGGTCATTATATGACAGAAATATTCTCAAAATTGTGT
TCTCAAATTTGTGTACCATCTTAACAATTGTTATTTTTACCATGTCTCTGAACCACCTATAC
=-====================
M13dir primer (direct strand):

```
    FILE NAME      39        48         58         68         78         88
98
    G3-1_M~2.SEQ
CTCCCGGCCGACCATGGCGGCCGCGGAGAAATACCAGGGACCCAAAACCCTGAGAAAAGA

||||||||||||||||||||||||||||
    INI.SEQ         .............
ATCACCAGGGACCCAAAACCCTGAGAAAAGA
                 -12        -3          7         17         27         37
47

    FILE NAME      99       108        118        128        138        148
158
    G3-1_M~2.SEQ
AACCCACATAGTTCATTTTTTCTTGGCTTCAGAGGTTACATCAATTTCCTCTGTAGGACA

||||||||||||||||||||||||||||||||||||||||||||||||||||||||||||
    INI.SEQ
AACCCACATAGTTCATTTTTTCTTGGCTTCAGAGGTTACATCAATTTCCTCTGTAGGACA
                   48        57         67         77         87         97
107

    FILE NAME     159       168        178        188        198        208
218
    G3-1_M~2.SEQ
TTTAAATTGGAACTATGGAATATTTTACATGAAACATAGATATAAATTTCAGTAGGGTTA

||||||||||||||||||||||||||||||||||||||||||||||||||||||||||||
    INI.SEQ
TTTAAATTGGAACTATGGAATATTTTACATGAAACATAGATATAAATTTCAGTAGGGTTA
                  108       117        127        137        147        157
167

    FILE NAME     219       228        238        248        258        268
278
    G3-1_M~2.SEQ
TAAAGACATCGATTCAGATAGTGTTTATGGTTATTCTGATAGTTACACCAAGGAGATTTA

||||||||||||||||||||||||||||||||||||||||||||||||||||||||||||
    INI.SEQ
TAAAGACATCGATTCAGATAGTGTTTATGGTTATTCTGATAGTTACACCAAGGAGATTTA
                  168       177        187        197        207        217
227

    FILE NAME     279       288        298        308        318        328
338
    G3-1_M~2.SEQ
TGATGGAGCAAAAATTCCAGCAACCCCCAAGAAAGCAACAGCAGCTGAAATTTCCAGTGC

||||||||||||||||||||||||||||||||||||||||||||||||||||||||||||
    INI.SEQ
TGATGGAGCAAAAATTCCAGCAACCCCCAAGAAAGCAACAGCAGCTGAAATTTCCAGTGC
```

```
                 228       237       247       257       267       277
287

    FILE NAME    339       348       358       368       378       388
398
    G3-1_M~2.SEQ
ATATTTCACATGGACTCTAGCCAGTAGATGGGAATATTATAATTCTAACTAAGTATTTCA

||||||||||||||||||||||||||||||||||||||||||||||||||||||||||||
    INI.SEQ
ATATTTCACATGGACTCTAGCCAGTAGATGGGAATATTATAATTCTAACTAAGTATTTCA
                 288       297       307       317       327       337
347

    FILE NAME    399       408       418       428       438
    G3-1_M~2.SEQ   CTCTTTGTATAAATATACAAAGAGCCTCTGTGGTATTCTCCCT
                   |||||||||| || ||||||||||||||||||||||||||||
    INI.SEQ        CTCTTTGTATGAACATACAAAGAGCCTCTGTGGTATTCTCCCT
                 348       357       367       377       387
-=-=
M13rev primer (complement):
    FILE NAME    619       628       638       648       658       668
678
    G3-1_M~1.SEQ
CCAGGATTGACACATCCTCTACTGTCCAAGAACATTTATACGGTGCTTCAGAGTCCTTGA

||||||||||||||||||||||||||||||||||||||||||||||||||||||||||||
    INI.SEQ
CCAGGATTGACACATCCTCTACTGTCCAAGAACATTTATACGGTGCTTCAGAGTCCTTGA
                 375       384       394       404       414       424
434

    FILE NAME    679       688       698       708       718       728
738
    G3-1_M~1.SEQ
CTTGGTGGAGTGGAGCTGTATTCACTCAATCATGTCATTTCTGACATTCTTGAAGAAAGC

||||||||||||||||||||||||||||||||||||||||||||||||||||||||||||
    INI.SEQ
CTTGGTGGAGTGGAGCTGTATTCACTCAATCATGTCATTTCTGACATTCTTGAAGAAAGC
                 435       444       454       464       474       484
494

    FILE NAME    739       748       758       768       778       788
798
    G3-1_M~1.SEQ
CTTGTTTAGGCCATCTCTCTCTGCGTCATAACCTTCATTGGAGTTTTAACAAAGCAAGCA

||||||||||||||||||||||||||||||||||||||||||||||||||||||||||||
    INI.SEQ
CTTGTTTAGGCCATCTCTCTCTGCGTCATAACCTTCATTGGAGTTTTAACAAAGCAAGCA
                 495       504       514       524       534       544
554

    FILE NAME    799       808       818       828       838       848
858
    G3-1_M~1.SEQ
AATGTTAGAATCAGTTAAAATGGTCTGTCTTGACCACATTAATGAAAAAAATCTAATTCA

||||||||||||||||||||||||||||||||||||||||||||||||||||||||||||
    INI.SEQ
AATGTTAGAATCAGTTAAAATGGTCTGTCTTGACCACATTAATGAAAAAAATCTAATTCA
                 555       564       574       584       594       604
614
```

```
    FILE NAME    859      868       878       888       898       908
918
    G3-1_M~1.SEQ
GATAATTCTTAATCAAAATGTCATCAGCGTTTAAAAATATTCAGGTCATTATATGACAGA

||||||||||||||||||||||||||||||||||||||||||||||||||||||||||||
    INI.SEQ
GATAATTCTTAATCAAAATGTCATCAGCGTTTAAAAATATTCAGGTCATTATATGACAGA
                 615      624       634       644       654       664
674

    FILE NAME    919      928       938       948       958       968
978
    G3-1_M~1.SEQ
AATATTCTCAAAATTGTGTTCTCAAATTTGTGTACCATCTTAACAATTGTTATTTTTACC

||||||||||||||||||||||||||||||||||||||||||||||||||||||||||||
    INI.SEQ
AATATTCTCAAAATTGTGTTCTCAAATTTGTGTACCATCTTAACAATTGTTATTTTTACC
                 675      684       694       704       714       724
734

    FILE NAME    979      988       998
    G3-1_M~1.SEQ   ATGTCTCTGAACCACCTATAC
                   |||||||||||||||||||||
    INI.SEQ        ATGTCTCTGAACCACCTATAC
                 735      744       754
```
