# Supplementary material for: Development of a Sensitive Digital Droplet PCR Screening Assay for the Detection of GPR126 Non-Coding Mutations in Bladder Cancer Urine Liquid Biopsies
Source: Biomedicines. 2023 Feb 8;11(2):495. doi: 10.3390/biomedicines11020495 (PMC9953558; doi:10.3390/biomedicines11020495)
Supplement: Supplementary file 1 [file biomedicines-11-00495-s001.zip › Figure S2_Jain.pdf]

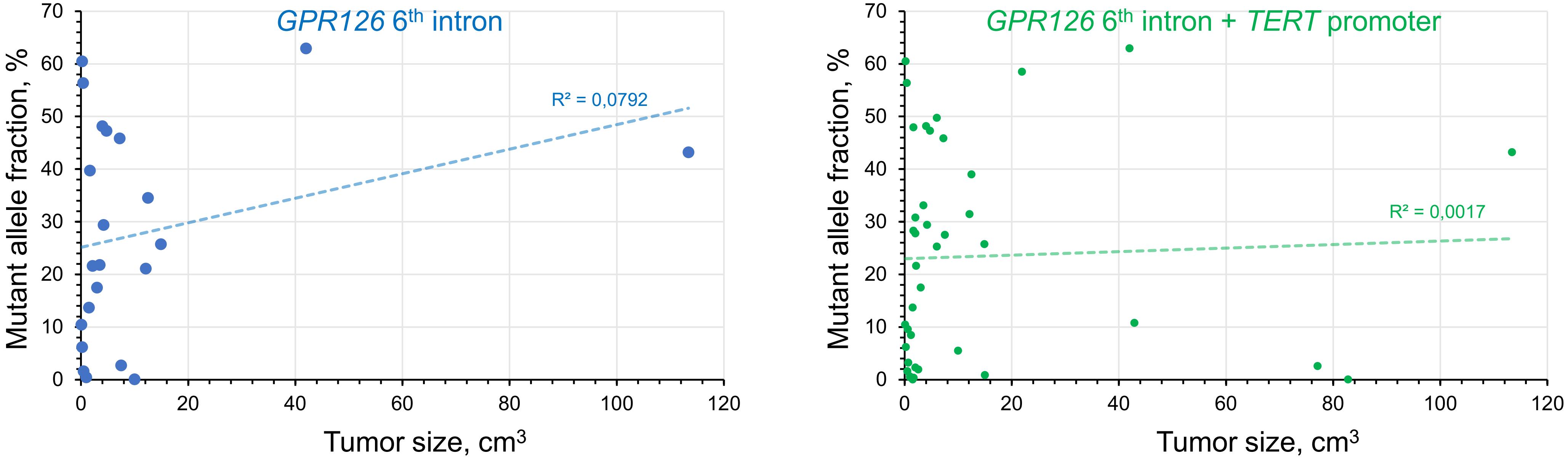

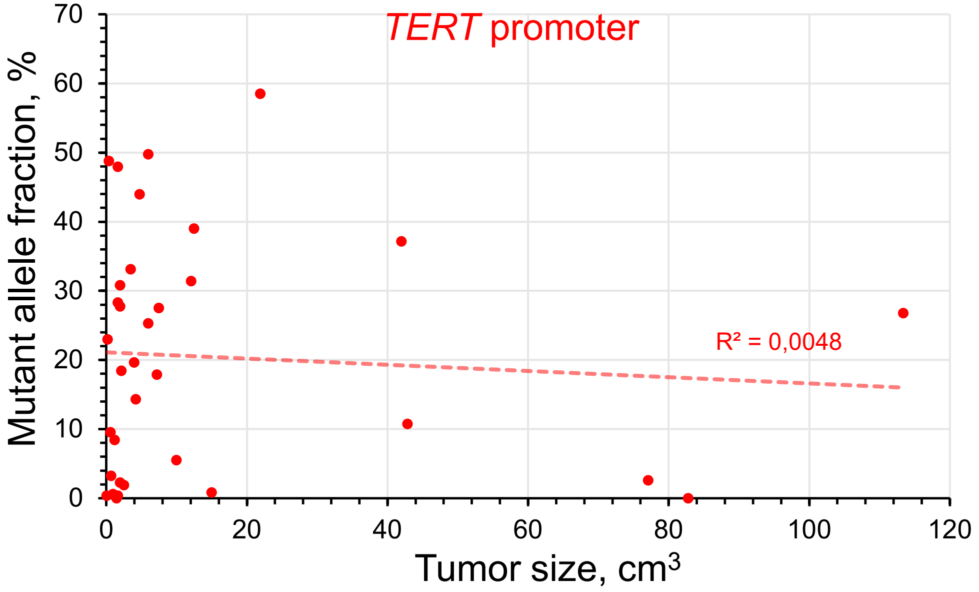

Figure S2. Scatter plots for mutant allele fraction and tumor size. Mutant allele fraction was assessed using digital droplet polymerase chain reaction on a QX200 AutoDG ddPCR System (Bio-Rad Laboratories, Inc.). Tumor size parameters were evaluated using transabdominal 3D ultrasonography on SonoAce X8 instrument (Samsung Medison Co., Ltd.). Correlation was not statistically significant in all cases ($p>0.05$). R^2 - coefficient of determination. Samples negative for the studied mutations were excluded.

GPR126 6th intron mutations: G/A substitution – chr. 6: 142,706,206; C/T substitution – chr. 6: 142,706,209 (assembly GRCh37). *TERT* promoter mutations: G/A substitution at -124 bp, chr. 5: 1,295,228; G/A substitution at -146 bp, chr. 5: 1,295,250 (assembly GRCh37).
