# Supplementary material for: Development of a Sensitive Digital Droplet PCR Screening Assay for the Detection of GPR126 Non-Coding Mutations in Bladder Cancer Urine Liquid Biopsies
Source: Biomedicines. 2023 Feb 8;11(2):495. doi: 10.3390/biomedicines11020495 (PMC9953558; doi:10.3390/biomedicines11020495)
Supplement: Supplementary file 1 [file biomedicines-11-00495-s001.zip › Figure S3_Jain.pdf]

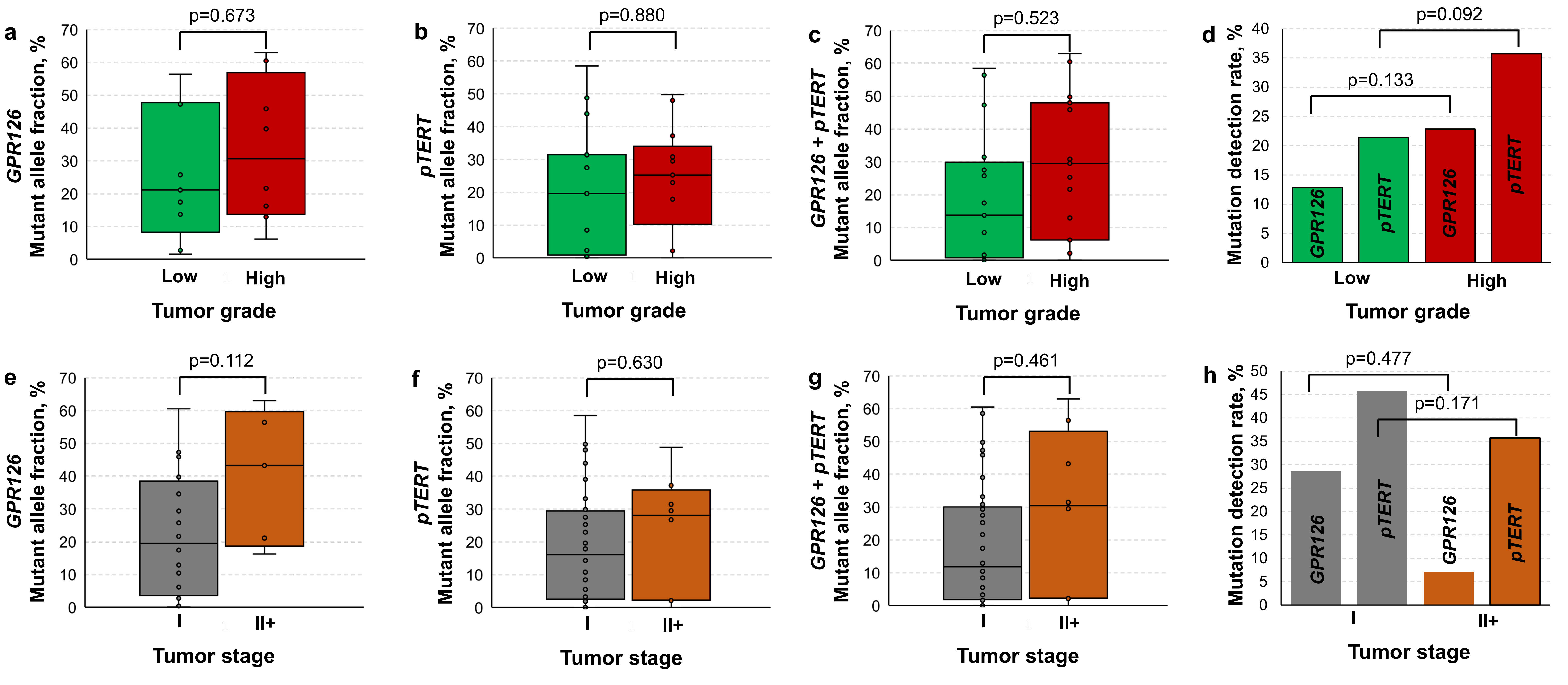
a
p=0.673
GPR126
Mutant allele fraction, %
70
60
50
40
30
20
10
0
Low
High
Tumor grade
b
p=0.880
pTERT
Mutant allele fraction, %
Low
High
Tumor grade
c
p=0.523
GPR126 + pTERT
Mutant allele fraction, %
Low
High
Tumor grade
d
p=0.092
p=0.133
Mutation detection rate, %
40
35
30
25
20
15
10
5
0
GPR126
pTERT
GPR126
pTERT
Low
High
Tumor grade
e
p=0.112
GPR126
Mutant allele fraction, %
I
II+
Tumor stage
f
p=0.630
pTERT
Mutant allele fraction, %
I
II+
Tumor stage
g
p=0.461
GPR126 + pTERT
Mutant allele fraction, %
I
II+
Tumor stage
h
p=0.477
p=0.171
Mutation detection rate, %
50
45
GPR126
pTERT
GPR126
pTERT
I
II+
Tumor stage

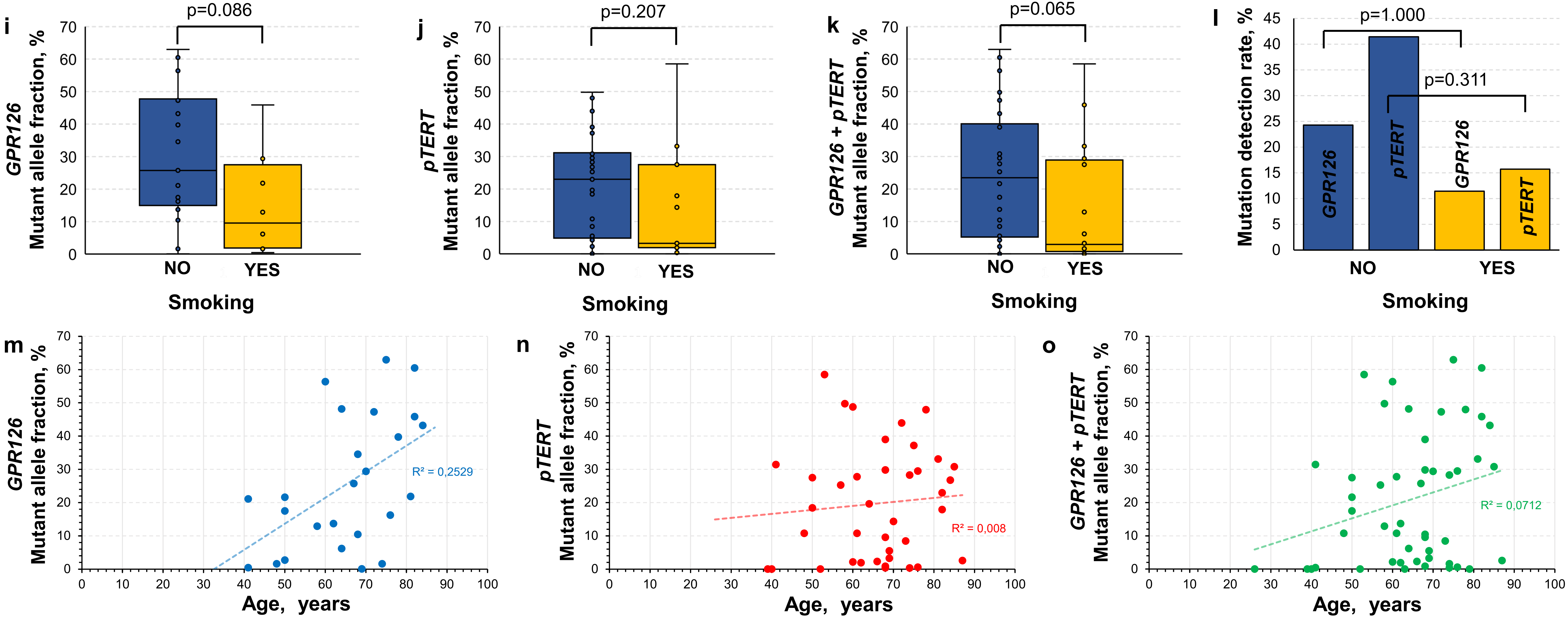


**Figure S3.** Comparison of mutant allele fractions and mutation detection rates with demographic and pathological characteristics of patients in the UBC group. **(a-d)** Box plots for tumor grades. **(e-h)** Box plots for tumor stages. Due to low incidence of stages II-IV, these cases were summarized as "II+" group. **(i-l)** Box plots for history of smoking. **(m-o)** Scatter plots for mutant allele fraction and age. Mutant allele fraction was assessed using digital droplet polymerase chain reaction on a QX200 AutoDG ddPCR System (Bio-Rad Laboratories, Inc). $R^2$ - coefficient of determination. Samples negative for the studied mutations were excluded from the quantitative comparisons (and for the assessment of correlation). *GPR126* 6$^{th}$ intron mutations: G/A substitution – chr. 6: 142,706,206; C/T substitution – chr. 6: 142,706,209 (assembly GRCh37). *TERT* promoter (*pTERT*) mutations: G/A substitution at -124 bp, chr. 5: 1,295,228; G/A substitution at -146 bp, chr. 5: 1,295,250 (assembly GRCh37).
