# Supplementary material for: Development of a Sensitive Digital Droplet PCR Screening Assay for the Detection of GPR126 Non-Coding Mutations in Bladder Cancer Urine Liquid Biopsies
Source: Biomedicines. 2023 Feb 8;11(2):495. doi: 10.3390/biomedicines11020495 (PMC9953558; doi:10.3390/biomedicines11020495)
Supplement: Supplementary file 1 [file biomedicines-11-00495-s001.zip › Figure S4_Jain.pdf]

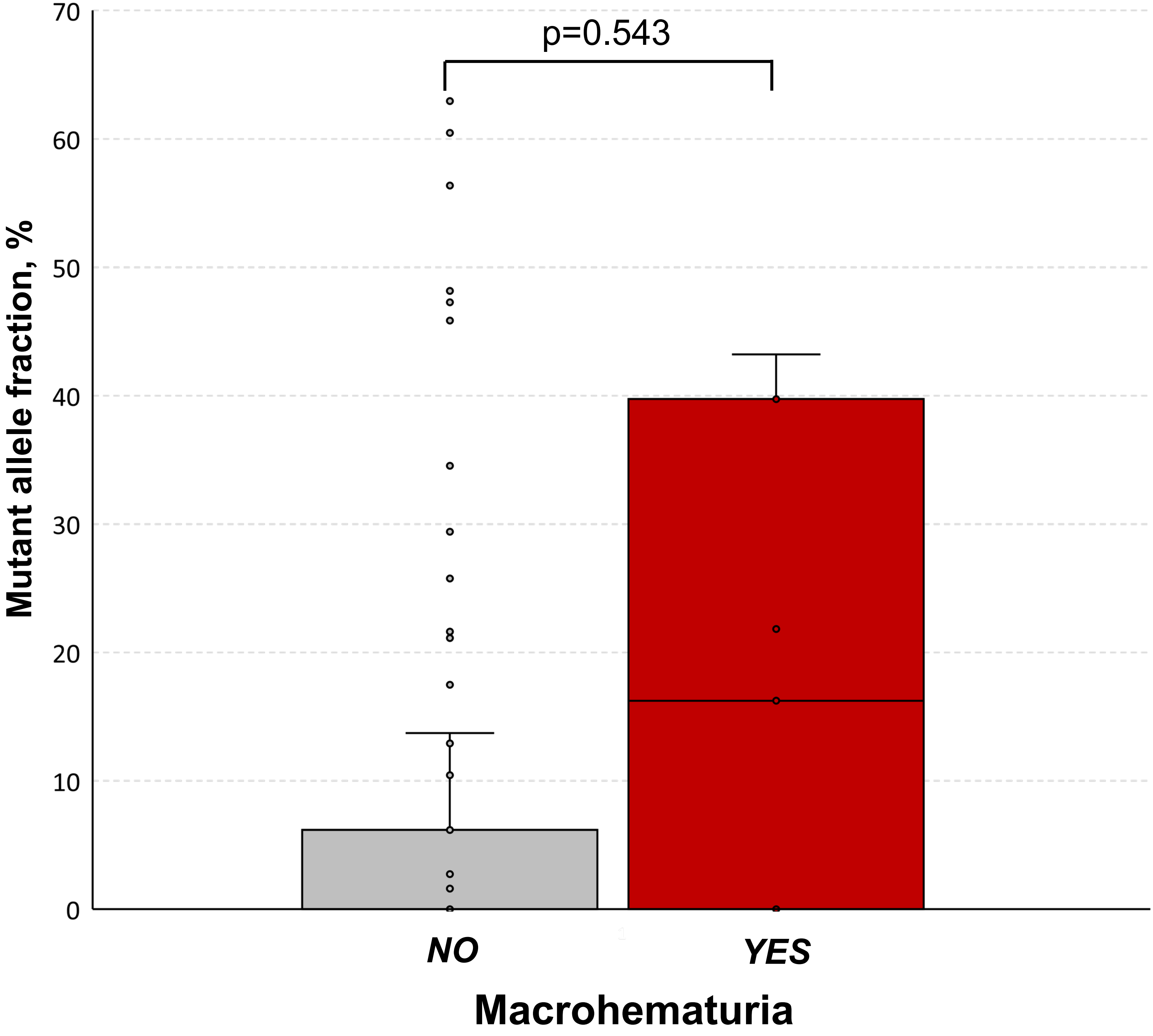

Figure S4. Impact of macrohematuria on the mutant allelic fraction of *GPR126* 6th intron. Mutant allele fraction was assessed using digital droplet polymerase chain reaction on a QX200 AutoDG ddPCR System (Bio-Rad Laboratories, Inc.). *GPR126* 6th intron mutations: G/A substitution – chr. 6: 142,706,206; C/T substitution – chr. 6: 142,706,209 (assembly GRCh37).
